# Postprandial glucose variability and clusters of sex hormones, liver enzymes, and cardiometabolic factors in a South African cohort of African ancestry

**DOI:** 10.1136/bmjdrc-2023-003927

**Published:** 2024-03-07

**Authors:** Bontle Masango, Julia H Goedecke, Michèle Ramsay, Karl-Heinz Storbeck, Lisa K Micklesfield, Tinashe Chikowore

**Affiliations:** 1 Division of Human Genetics, National Health Laboratory Service (NHLS), School of Pathology, University of the Witwatersrand, Faculty of Health Sciences, Johannesburg, South Africa; 2 South African Medical Research Council/University of the Witwatersrand, Developmental Pathways for Health Research Unit (DPHRU), University of the Witwatersrand, Faculty of Health Sciences, Johannesburg, South Africa; 3 Biomedical Research and Innovation Platform, South African Medical Research Council, Cape Town, South Africa; 4 Sydney Brenner Institute for Molecular Bioscience, University of the Witwatersrand, Faculty of Health Sciences, Johannesburg, South Africa; 5 Department of Biochemistry, Stellenbosch University, Stellenbosch, South Africa; 6 Harvard Medical School, Boston, Massachusetts, USA; 7 Channing Division of Network Medicine, Brigham and Women's Hospital, Boston, Massachusetts, USA

**Keywords:** Type 2 Diabetes, Postprandial Glucose, Metabolism, Lipids

## Abstract

**Introduction:**

This study aimed to, first, determine the clusters of sex hormones, liver enzymes, and cardiometabolic factors associated with postprandial glucose (PPG) and, second to evaluate the variation these clusters account for jointly and independently with polygenic risk scores (PRSs) in South Africans of African ancestry men and women.

**Research design and methods:**

PPG was calculated as the integrated area under the curve for glucose during the oral glucose tolerance test (OGTT) using the trapezoidal rule in 794 participants from the Middle-aged Soweto Cohort. Principal component analysis was used to cluster sex hormones, liver enzymes, and cardiometabolic factors, stratified by sex. Multivariable linear regression was used to assess the proportion of variance in PPG accounted for by principal components (PCs) and type 2 diabetes (T2D) PRS while adjusting for selected covariates in men and women.

**Results:**

The T2D PRS did not contribute to the PPG variability in both men and women. In men, the PCs’ cluster of sex hormones, liver enzymes, and cardiometabolic explained 10.6% of the variance in PPG, with PC1 (peripheral fat), PC2 (liver enzymes and steroid hormones), and PC3 (lipids and peripheral fat) contributing significantly to PPG. In women, PC factors of sex hormones, cardiometabolic factors, and liver enzymes explained a similar amount of the variance in PPG (10.8%), with PC1 (central fat) and PC2 (lipids and liver enzymes) contributing significantly to PPG.

**Conclusions:**

We demonstrated that inter-individual differences in PPG responses to an OGTT may be differentially explained by body fat distribution, serum lipids, liver enzymes, and steroid hormones in men and women.

**WHAT IS ALREADY KNOWN ON THIS TOPIC**

- There is high inter-individual variability in postprandial glucose (PPG) in response to identical meals. High PPG levels can be a risk factor for cardiovascular diseases and type 2 diabetes (T2D), even in individuals with normal glucose tolerance.

**WHAT THIS STUDY ADDS**

- The precise sex hormones, liver enzymes, and cardiometabolic factors that contribute to PPG variability in South Africans of African ancestry are not known. Our study identified factors (body fat distribution, serum lipids, liver enzymes, and steroid hormones) that predict PPG variability in South African middle-aged African ancestry individuals using principal component clustering on an extensive range of phenotypic data.

**HOW THIS STUDY MIGHT AFFECT RESEARCH, PRACTICE, OR POLICY**

- This is the first study in Africa to explore factors associated with PPG variability and this information is key to our understanding of the pathophysiology of T2D in the Sub-Saharan Africa region which has the highest projected rates of increase in diabetes. Also, characterizing the factors that contribute to inter-individual variability in PPG is the first step toward risk stratification and implementing future precision medicine approaches for people in Africa.

## Introduction

Type 2 diabetes (T2D) is a global healthcare burden affecting approximately 463 million individuals worldwide, with 19.4 million from Africa in 2019.^1^ The Sub-Saharan Africa (SSA) region has the highest projected rates of increase in diabetes (142.9%) compared with the other International Diabetes Federation regions.^1^ SSA has limited resources to manage the burden, and as a result, it has a high proportion (59.7%) of undiagnosed people living with diabetes calculated using the random-effect generalized linear regression.^1^ There is a need for cost-effective risk stratification tools that allow for early detection to delay and prevent the progression of T2D.

Postprandial glucose (PPG) refers to the blood glucose levels after a meal and is a measure of diabetes risk.^2^ PPG is assessed using the oral glucose tolerance test (OGTT), a technique widely used in clinical settings to diagnose T2D.^3^ Studies have examined PPG variability from an OGTT using the shape index^3^ and the integrated area under the curve (iAUC) calculated from the trapezoidal method.^4^ The shape index gives relevant information about different metabolic risk profiles^3^; however, it only uses the 90- and 120-minute glucose readings and does not account for the total variability in the OGTT.^3^ The iAUC method indicates whole glucose variability and glycemic control.^5^ It considers the changes in the entire glucose curve after glucose load and provides more information about glucose tolerance status.^6^

PPG regulation is closely linked to insulin secretion, insulin resistance, and beta-cell function, all of which play crucial roles in the development of T2D.^7^ The measure of PPG provides insight into the inter-individual variability in response to identical meals and is evaluated to assess the ability to regulate glucose metabolism and insulin sensitivity.^8^ Understanding the determinants of PPG variability is essential to determine factors that can be targeted in interventions for preventing and delaying the progression of T2D. Further, the determinants of PPG can be used to determine variability in dietary responses, which can assist with risk stratification, and the implementation of future precision medicine approaches for people in Africa,^2^ which have not been previously explored.

Several risk factors are thought to affect PPG variability. These include genetics, meal composition and context, lifestyle factors such as physical activity, sedentary behavior and sleep, body composition and fat distribution, health status, and medication.^9^ However, these factors do not act in isolation. Hence, cluster analysis of demographic, anthropometric, lifestyle, and biochemical measures has been suggested to reveal important determinants of postprandial variability.^9^ Studies have used cluster and principal component analysis (PCA) to group cardiometabolic variables. Their findings illustrated the ability of cluster analysis to stratify participants based on their metabolites and determine how postprandial metabolism reflects an individual’s cardiometabolic health status.^10 11^ Although cluster analysis is a fundamental approach, the technique has only been performed in the European context and is yet to be evaluated among continental Africans.

Variations in the genes related to PPG can indirectly affect T2D risk by influencing glucose metabolism and insulin response. Evidence exists of genetic factors that influence PPG variability.^12^ In a heritability study of twins, genetic factors accounted for approximately 48% of the variation in PPG.^2^ While the study showed the proportion of the variation accounted for by genetic factors, the specific genetic factors contributing to the PPG variation are still unknown. Genome-wide association studies (GWAS) have helped to identify specific genetic variants in Europeans.^12^ For example, a European study found that only 9% of the variation in PPG could be accounted for by specific genetic factors and also associated with T2D risk.^2^ To our knowledge, no similar studies have been done in Africa, and GWAS are still limited. This highlights the need for more studies to determine genetic variants associated with PPG variability in Africa. Ongoing research evaluates polygenic risk score (PRS) for T2D in Africa.^13^ The research involves identifying and analyzing genetic variants associated with T2D risk and then using this information to calculate PRS for individuals within the African population. Although this is a good approach, it is not known if the PRS for T2D in African populations is associated with PPG variability, which is an important risk factor for T2D.

Further, no studies in African populations have evaluated the combined impact of anthropometric, lifestyle, biochemical measures, sex hormones, cardiometabolic, and genetic factors on PPG variability. Accordingly, our study aimed to assess the contribution of T2D PRS, and anthropometric, lifestyle, biochemical measures, sex hormones, and cardiometabolic factors associated with PPG variability in South Africans of African ancestry men and women.

## Research design and methods

This is a cross-sectional study combining data from the Africa Wits-IN-DEPTH partnerships for Genomic Research (AWI-Gen)^14^ and Middle-aged Soweto Cohort (MASC),^15^ the former provided genotype and the latter provided phenotype data on the same participants. MASC does not differ in terms of age, sex, sociodemographic, or lifestyle factors from the AWI-Gen cohort and is therefore representative of an urban African ancestry population.

### Study participants

The same participants in this study were part of both MASC and AWI-Gen. Baseline data were collected between 2011 and 2014 as part of AWI-Gen which is a Human Heredity and Health in Africa (H3A) Consortium study,^16^ from which a subsample of participants were randomly selected in 2017/2018 (n=1112). Data collection occurred at the South African Medical Research Council/University of the Witwatersrand, Developmental Pathways for Health Research Unit at the Chris Hani Baragwanath Hospital in Soweto, Johannesburg, South Africa.

**Figure 1:**
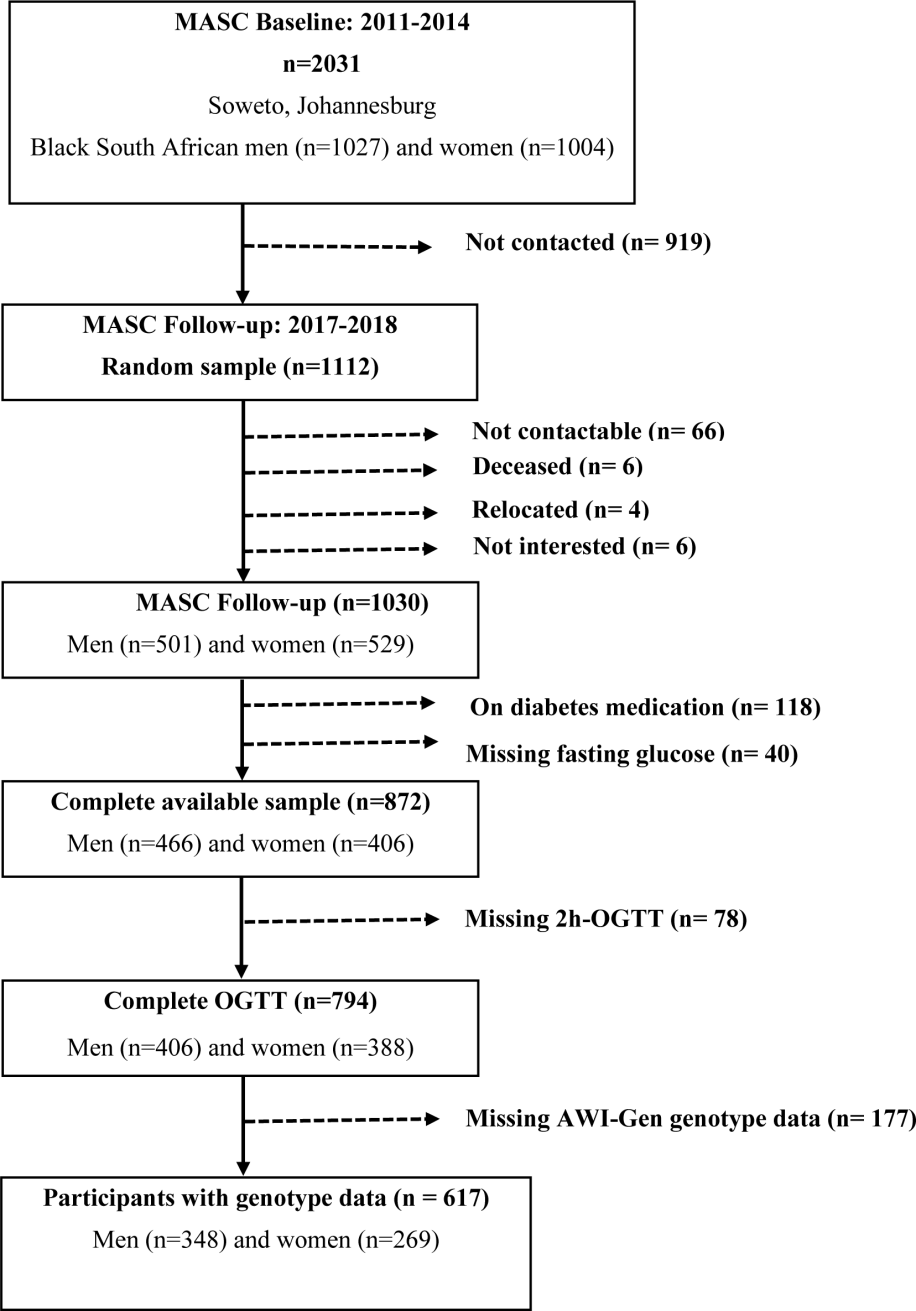
Consolidated Standards of Reporting Trials (CONSORT) diagram for the Middle-aged Soweto Cohort (MASC) postprandial glucose (PPG) variability study. AWI-Gen, Africa Wits-IN-DEPTH partnerships for Genomic Research; OGTT, oral glucose tolerance test.

The inclusion criteria for this PPG variability study were men and women in MASC with complete OGTT data from the follow-up study (n=794; n=406 men and n=388 women) and genotype data from the AWI-Gen study (n=617). The following exclusion criteria were applied: participants with known diabetes or those taking diabetes medication, missing fasting glucose measurements, and incomplete OGTT (figure 1).

The MASC study was conducted following the Declaration of Helsinki and was approved by the Human Research Ethics Committee (Medical) of the University of the Witwatersrand (clearance certificate no M160604 and M160975). Before participation in the research trial, all participants provided informed consent. AWI-Gen study ethical clearance was approved by the Human Research Ethics Committee (Medical) of the University of the Witwatersrand (Wits) (protocol numbers M121029 and M170880). This PPG variability study was approved by the Human Research Ethics Committee (Medical) of the University of the Witwatersrand (clearance certificate no M220296).

### OGTT and PPG

A standard 2-hour OGTT was then conducted. After ingesting 75 g of glucose (dissolved in 250 mL water), blood samples were drawn at 30-minute intervals for 2 hours to measure plasma glucose concentrations. PPG was calculated in R (V.2022.02.0) using the trapezoidal rule.^17^

### Socio-demographic factors and health questionnaires

Demographic factors, socioeconomic status, lifestyle, and medical history were collected through interviewer-administrated questionnaires and captured on the Research Electronic Data Capture (REDCap) tool.^18^ This included age, sex, marital status (married/cohabiting vs unmarried/not cohabiting), the highest education level attained (primary, secondary or tertiary), current alcohol consumption (no alcohol intake or alcohol intake (monthly or less and two to four times a month) or alcohol intake (two to three times per week or more), smoke tobacco (yes or no), and employed. Asset index was calculated by summing assets reported to be owned in the household.^19^ HIV status was assessed using an antibody HIV test in participants not living with HIV (Wondfo One Step HIV–1/2 Whole Blood/Serum/Plasma: Test 2 lines Guangzhou Wondfo Biotech, Hong Kong, China). Those who tested positive for HIV were referred to a local clinic and were retained in the study. In addition, participants were asked to bring in their chronic medications, which were documented, for example, medications for diabetes, hypertension, dyslipidemia, and hormone replacement therapy (yes or no). The menstrual stage was assessed in women using the final menstrual cycle; women were classified as premenopausal, perimenopausal, or postmenopausal.^20^

### Body composition, body fat distribution, and blood pressure measures

Participants’ weight and height were measured to the nearest 0.1 kg and 0.1 cm, using a digital scale and wall-mounted stadiometer, respectively.^15 16^ The waist circumference (WC) and hip circumference (HC) were measured to the nearest 0.1 cm with a non-stretchable tape. For WC the tape was placed horizontally mid-way between the iliac crest in the mid-axillary plane and the lowest rib margin and for HC it was placed around the level of the most significant protrusion of the buttocks. The waist-to-hip ratio (WHR) was calculated. Subtotal (total body minus head) fat mass, fat-free soft tissue mass (FFSTM), and regional adiposity were measured using dual-energy X-ray absorptiometry (DXA) (QDR 4500A, Hologic, Bedford, New York, USA, APEX software V.4.0.2). Regional adiposity, which includes trunk, arm, gynoid, android, and leg fat mass, was expressed as a percentage of subtotal fat mass (%FM). Abdominal visceral adipose tissue (VAT) and subcutaneous adipose tissue (SAT) were estimated as described before.^21^ Systolic and diastolic blood pressures were measured to the nearest 0.1 mm Hg on the left arm with a digital blood pressure monitor (Omron M6, Kyoto, Japan) and appropriate cuffs. After the participant had been seated for at least 5 min, three blood pressure readings were taken at 2-minute intervals. For each participant, the average of the second and third readings was used in the analyses.

### Physical activity and sleep

Participants were asked to wear the accelerometers for 7 days and nights to assess their physical activity and they were also asked to record their daily sleep (including naps) and waking times in a dairy. The variables (physical activity and sleep) were reported as total movement volume (Euclidean norm minus one expressed as milligravitational units) and time (in minutes per day) spent sleeping.^21^

### Fasting blood sampling and biochemical measures

At ~08:00 and after an overnight (10–12 hours) fast, blood samples were taken for the measurement of fasting insulin, glucose, liver enzymes (alanine transaminase (ALT), aspartate transaminase (AST), and gamma-glutamyl transferase (GGT)), gonadotrophins (luteinizing hormone (LH), follicle-stimulating hormone (FSH)), sex hormones (testosterone, estrogen, androstenedione, and dehydroepiandrosterone (DHEA)), lipids (total cholesterol (TC), high-density lipoprotein cholesterol (HDL-C), low-density lipoprotein cholesterol (LDL-C), and triglyceride (TG)), and glucocorticoids (cortisol and cortisone).

FSH and LH were measured with the ARCHITECT Chemiluminescent Microparticle Immunoassay assay (Abbott Laboratories). Sex steroids and glucocorticoids were measured by ultra-high performance liquid chromatography–tandem mass spectrometry.^22^ Serum insulin and lipid concentrations were measured with Immulite 1000 Immunoassay System (Siemens Chemiluminescent Healthcare GmbH, Henkestr, Germany) and glucose concentrations with a Randox RX Daytona Chemistry Analyzer (Randox Laboratories, London, UK), respectively. TC, TG, and HDL-C were measured directly from the Immulite 1000 Immunoassay System (Siemens Chemiluminescent Healthcare GmbH). LDL-C was calculated with the Friedewald equation.^23^

### Classification of OGTT

Participants were classified as normal glucose tolerance (NGT, fasting glucose <6.1 mmol/L and 2-hour OGTT <7.8 mmol/L), impaired glucose metabolism (fasting glucose: 6.1–6.9 mmol/L and 2-hour postglucose load: 7.8–11.0 mmol/L), and T2D (fasting glucose ≥7 mmol/L and 2-hour postglucose load ≥11.1 mmol/L, or taking T2D medication) according to the WHO criteria.^24^

### Genotyping and imputation

The 617 MASC participants with complete OGTT data were genotyped using the 2.3 M Single Nucleotide Polymorphism (SNP) H3Africa array at Illumina FastTrack Microarray services (Illumina, San Diego, California, USA) and the Illumina pipeline was used for genotype calling.^25^ The preimputation and imputation quality control were carried out using the H3ABioNet/H3Agwas pipeline and the African Genome Resources reference panel (Eagle2+PBWT (Positional Burrows–Wheeler Transform) pipeline) at the Sanger Imputation Server.

### Statistical analysis

#### Descriptive statistics

Data were analyzed using R V.4.3.1. Categorical variables are represented as frequencies (n) and percentages (%), continuous variables that were normally distributed are represented as mean±SD, and continuous variables that were not normally distributed are represented as median (25th–75th percentiles). A Shapiro-Wilk test was used to check if continuous variables followed a normal distribution. Differences between men and women were assessed using Mann-Whitney U and t-tests for non-parametric and parametric continuous variables, respectively. The χ^2^ test was used to compare categorical variables between men and women.

#### Multivariable analysis

Due to the significant differences in lifestyle, anthropometric, biochemical measures, and cardiometabolic factors, as well as the differential association between body fatness and T2D risk,^26^ separate PCAs were performed for men and women. PCA was used for multivariable dimension reduction to derive latent variables termed principal components (PCs) from 31 anthropometric, lifestyle, biochemical measures, sex hormones, and cardiometabolic factors for men and women separately. PCA was chosen because it is a data-driven approach that can calculate the covariances of data, find patterns in the data, and reduce the dimension of large datasets.^27^ The following variables were included in the PCA: central fatness (WC, SAT, android fat, and trunk fat), peripheral fatness (HC, gynoid fat, and leg fat), liver enzymes (AST, GGT, and ALT), serum lipids (TG, TC, HDL and LDL), gonadotrophins (FSH and LH), sex hormones (testosterone, estradiol, androstenedione, and DHEA), and glucocorticoids (cortisol and cortisone). These variables were standardized to ensure they were on the same scale before the PCA. The scree plot was used to determine the number of PCs to retain (online supplemental figure 1). The PCs were named based on the variables that had higher factor loadings.

10.1136/bmjdrc-2023-003927.supp1 Supplementary data

#### Polygenic risk scores

We used a PRS developed and validated for T2D in African-ancestry populations.^13^ This PRS was developed using PRSice 2 software and computed using the following parameters for pruning (clumping distance of 500 kb and R^2^ of 0.5) and using the African American GWAS from the Million Veterans Program as the discovery dataset while adjusting for age, sex, and population structure as described elsewhere.^13^ For our study, we used the summary statistics of previously computed and validated T2D PRS to compute the PRS for the participants using PRSice in the 617 MASC participants.^13^ This PRS was then used in the linear models described below.

#### Linear regression models

Multivariable linear regression was used to evaluate the independent contribution of T2D PRS and PCs (sex hormones, liver enzymes, and cardiometabolic factors) to PPG variability, in men and women separately. Three separate regression models were performed to determine PPG variability. Null model included covariates that are known to be associated with glycemic response to glucose: age, menopausal stage, smoking status, alcohol consumption, blood pressure medications, lipid medication, HIV status, total physical activity, total sleep, and asset index. Genetic principal components for T2D PRS were added to Models 2 and 3 to correct for population structure; Model 1: null model and PCs (anthropometric, lifestyle, biochemical measures, sex hormones, and cardiometabolic factors); Model 2: null model and T2D PRS; Model 3: null model, T2D PRS, and PCs (anthropometric, lifestyle, biochemical measures, sex hormones, and cardiometabolic factor). The variance (R^2^) from the models enabled us to determine the relative contribution of risk PCs, T2D PRS, and these two combined toward PPG variability.

## Results

### Participants’ sociodemographic and body fat distribution

A total of 794 participants with a mean age of 53 (6.3) years were included (table 1). More men were married than women (52.8% vs 41.9%), more men smoked tobacco (50.9% vs 6.2%) and consumed alcohol (33.6% vs 5.2%) compared with women. HIV status did not differ by sex.

**Table 1:** Sociodemographic status, body composition, and biochemical measures in South African men and women

Variable	Men	Women	P value
n (%)	406 (51.1)	388 (48.9)	–
Sociodemographic characteristics			
Age (years)	52.0 (47.0–58.0)	53.4 (49.0–58.0)	0.112
Married/cohabiting (n (%))	214 (52.8)	162 (41.9)	<0.001
Employed (n (%))	163 (40.2)	153 (39.5)	0.895
Lifestyle characteristics			
Sleep (min/d) (n=599)	424.0 (371.7–471.7)	411.8 (362.2–455.65)	0.059
ENMO (milli-g) (n=600)	14.3 (11.4–17.5)	12.3 (10.0–14.5)	<0.001
Smoking tobacco (n (%))	206 (50.9)	24 (6.2)	<0.001
HIV positive (n (%)))	72 (20.9)	47 (17.5)	0.586
Alcohol intake (n (%))			<0.001
No alcohol intake	103 (25.4)	277 (71.6)	
Alcohol intake (monthly or less and 2–4 times a month)	166 (41.0)	90 (23.3)	
Alcohol intake (2–3 times per week or more)	136 (33.6)	20 (5.2)	
Education (n (%))			0.204
No formal schooling/elementary school level (n (%))	47 (33.3)	31 (8.7)	
Secondary school level (n (%))	287 (17.6)	311 (79.8)	
Tertiary education (n (%))	135 (48.9)	45 (11.3)	
Body composition measures			
Height (cm)	171.4 (167.0–175.2)	158.2 (154.0–162.3)	<0.001
Weight (kg)	72.2 (60.8–84.2)	80.9 (71.7–94.1)	<0.001
BMI (kg/m^2^)	24.5 (20.8–29.1)	32.8 (28.5–37.3)	<0.001
Waist circumference (cm)	92.4 (82.0–102.7)	95.5 (87.4–103.6)	0.003
Hip circumference (cm)	98.1 (90.8–105.4)	114.6 (106.4–123.4)	<0.001
WHR	0.94 (0.90–0.98)	0.85 (0.77–0.88)	<0.001
DXA-derived body composition (n=756)			
Fat-free soft tissue mass (kg)	47.5 (41.6–53.1)	40.4 (36.4–44.9)	<0.001
Whole body fat mass (kg)	17.4 (11.6–22.8)	35.0 (28.5–41.7)	<0.001
Body fat (%)	26.0 (20.7–30.4)	45.2 (41.6–48.4)	<0.001
Trunk (%FM)	46.6±5.1	43.3±5.9	<0.001
Leg (%FM)	41.0±4.8	44.2±6.3	<0.001
Arm (%FM)	12.4±1.3	12.5±1.8	0.738
Gynoid (%FM)	17.1±1.8	17.8±2.3	<0.001
Android (%FM)	8.4±1.5	7.4±1.4	<0.001
VAT (cm^2^)	85.5±43.3	104.1±43.3	<0.001
SAT (cm^2^)	197.0±119.7	461.4±145.8	<0.001
VAT/SAT	0.51 (0.36–0.66)	0.23 (0.17–0.27)	<0.001
Glucose and insulin measures (n=794)			
Fasting glucose (mmol/L)	5.0 (4.4–5.5)	5.0 (4.5–5.4)	0.911
2-hour glucose (mmol/L)	7 (4.4–6.8)	5.8 (4.9–7.1)	0.017
PPG (mmol/L)	13.0 (11.0–14.8)	12.4 (10.7–14.8)	0.042
Glucose tolerance status n (%)			0.237
NGT (FPG <6.1/2-hour PG <7.8 mmol/L)	331 (81.5)	308 (79.6)	
IGM (FPG 6.1–6.9/IGT 2-hour PG: 7.8–11.0)	56 (13.8)	61 (15.8)	
T2DM (FPG ≥7/2-hour PG ≥11.1 mmol/L)	19 (4.7)	18 (4.7)	
Serum lipids			
TG (mmol/L) (n=793)	0.9 (0.7–1.4)	0.8 (0.6–1.0)	<0.001
TC (mmol/L) (n=793)	4.3 (3.6–4.9)	4.5 (3.9–5.2)	0.003
LDL-C (mmol/L) (n=793)	2.4 (1.8–3.0)	2.8 (2.2–3.4)	<0.001
HDL-C (mmol/L) (n=793)	1.3 (1.0–1.5)	1.3 (1.1–1.5)	0.208
Liver enzymes			
ALT (μKat/L) (n=793)	0.4 (0.3–0.5)	0.3 (0.3–0.4)	<0.001
AST (μKat/L) (n=793)	0.7 (0.5–0.9)	0.7 (0.6–0.8)	<0.001
GGT (μKat/L) (n 793)	0.7 (0.5–1.3)	0.5 (0.3–0.7)	<0.001
Sex hormones			
Estrogen (pmol/L) (n=767)	125.5 (96.0–154.5)	88.0 (57.0–135.9)	<0.001
Testosterone (nM) (n=774)	15.3 (11. 9–19.5)	0.3 (0.2–0.5)	<0.001
Gonadotrophins			
LH (pmol/L) (n=768)	3.6 (2.5–5.0)	18.6 (10.4–19.4)	<0.001
FSH (pmol/L) (n=768)	4.9 (3.5–7.4)	46.5 (23.5–64.0)	<0.001
Steroid hormones			
Androstenedione (nmol/L) (n=774)	2.6 (1.8–3.5)	1.3 (0.9–1.8)	<0.001
DHEA (nmol/L) (n=774)	1.8 (0.0–8.9)	5.2 (1.8–7.9)	<0.001
Cortisol (pmol/L) (n=774)	322.8 (246.0–412.3)	250.9 (194.5–321.7)	<0.001
Cortisone (pmol/L) (n=774)	60.7 (51.1–69.7)	46.1 9 (39.3–55.5)	<0.001

Values are expressed as mean±SD, median (25th–75th percentile), or n (%).

ALT, alanine transaminase; AST, aspartate transaminase; BMI, body mass index; DHEA, dehydroepiandrosterone; DXA, dual-energy X-ray absorptiometry; ENMO, Euclidean norm minus one; FPG, fasting plasma glucose; FSH, follicle-stimulating hormone; GGT, gamma-glutamyl transferase; HDL-C, high-density lipoprotein cholesterol; IGM, impaired glucose metabolism; IGT, impaired glucose tolerance; LDL-C, low-density lipoprotein cholesterol; LH, luteinizing hormone; NGT, normal glucose tolerance; PG, plasma glucose; PPG, postprandial glucose; SAT, subcutaneous adipose tissue; TC, total cholesterol; T2DM, type 2 diabetes mellitus; TG, triglycerides; VAT, visceral adipose tissue; WHR, waist-to-hip ratio.

The mean BMI was higher in women than men (p<0.001), and more women were classified as living with obesity than men (92.5% vs 20.9%) (table 1). Accordingly, women had significantly higher waist and hip circumferences, but the WHR was lower in women than in men. The FFSTM was higher in men (p<0.001), but body fat (%) and whole body fat mass (kg) were higher in women than in men. When expressed relative to whole body FM, men had more central fat mass (trunk FM and android FM), while women had more fat in the lower extremities (leg FM and gynoid FM). Arm FM did not differ between the sexes. The central measures, VAT and SAT (both p<0.001), were higher in women, but the VAT/SAT ratio was higher in men than in women (table 1).

### Biochemical measures and PPG

**Figure 2:**
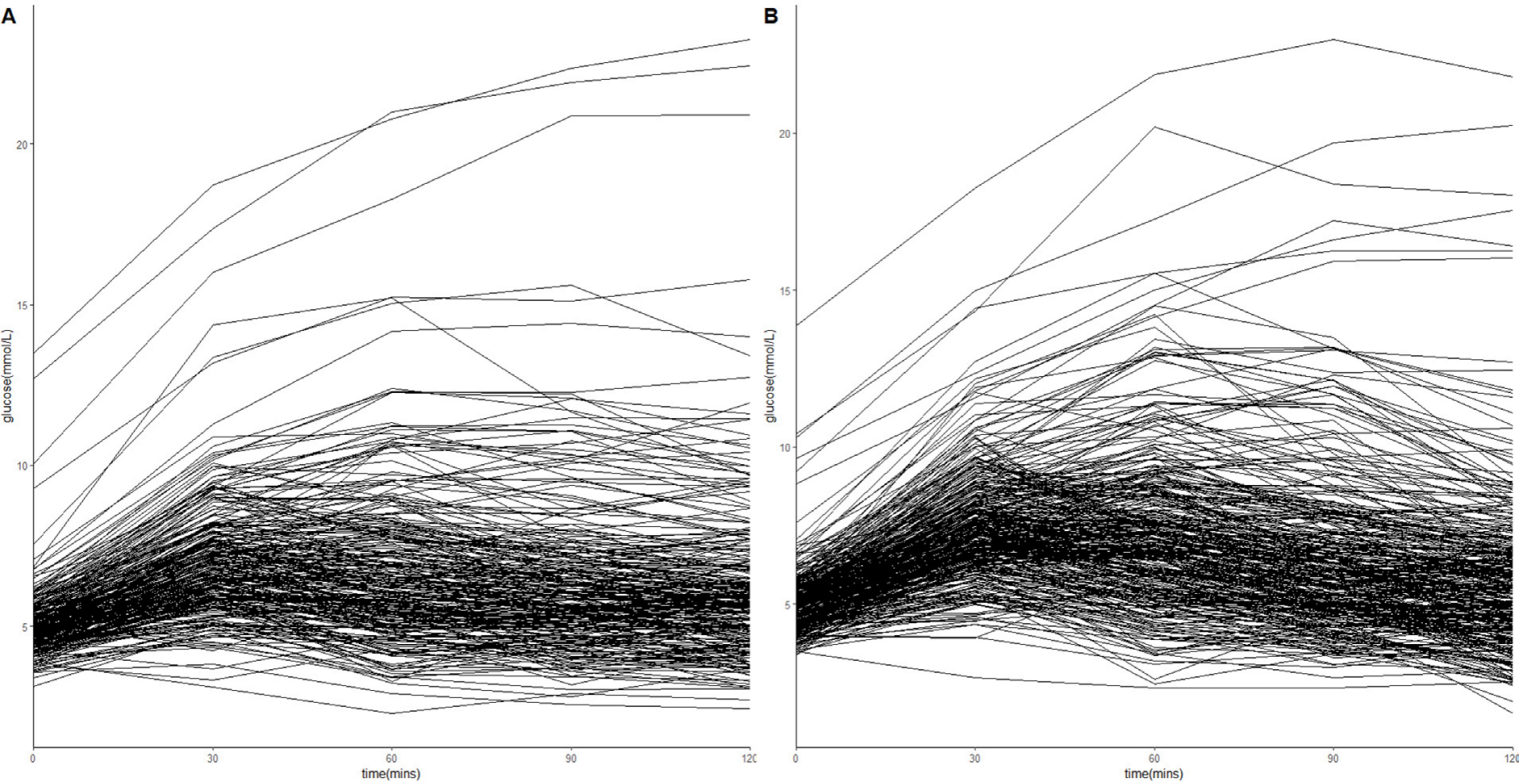
Glucose curves showing postprandial glucose inter-individual variability in women (A) and men (B) during a 2-hour oral glucose tolerance test.

Fasting glucose levels and glucose tolerance categories did not differ between men and women. Although PPG was slightly higher in men compared with women (p=0.042) (table 1), there were still considerable inter-individual differences in PPG responses to the OGTT in both men and women (figure 2).

While HDL concentrations were similar, TC and LDL concentrations were higher and TG lower in women compared with men. The liver enzymes AST, ALT, and GGT were higher in men than in women (p<0.001). The gonadotrophins (LH and FSH) were higher in women than men. DHEA was higher in women, whereas androstenedione, estrogen, and testosterone were higher in men. Cortisol and cortisone were higher in men than in women.

### Principal component analysis

**Figure 3:**
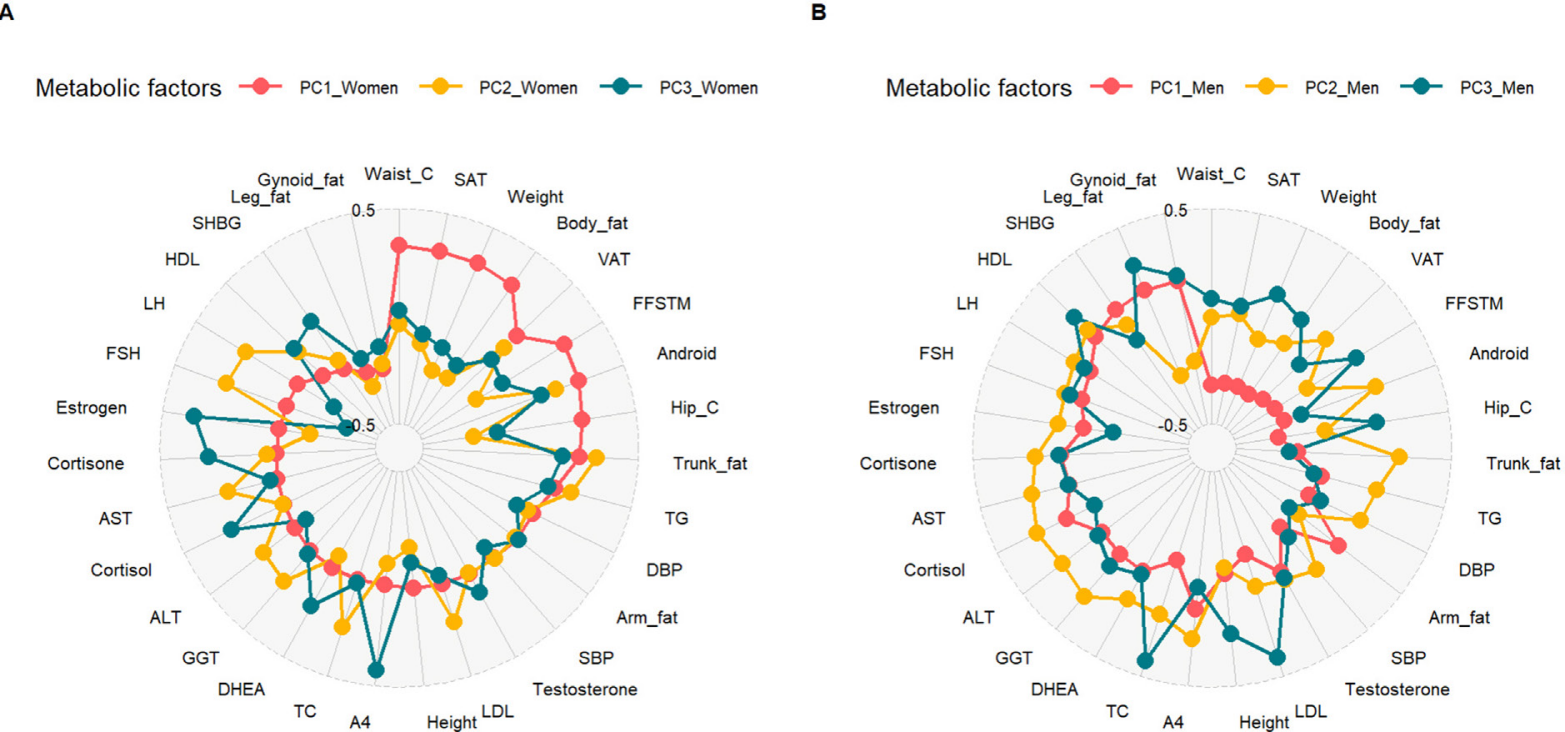
Radar plot showing factor loadings contributions of the risk factors to the principal components in women (A) and men (B). ALT, alanine transaminase; AST, aspartate transaminase; DBP, diastolic blood pressure; DHEA, dehydroepiandrosterone; DXA, dual-energy X-ray absorptiometry; FSH, follicle-stimulating hormone; GGT, gamma-glutamyl transferase; HDL-C, high-density lipoprotein cholesterol; LDL-C, low-density lipoprotein cholesterol; LH, luteinizing hormone; SAT, subcutaneous adipose tissue; SBP, systolic blood pressure; TC, total cholesterol; TG, triglycerides; VAT, visceral adipose tissue; SHBG, sex hormone-binding globulin; FFSTM, fat-free soft tissue mass.

Based on the differences in characteristics between men and women (table 1), separate PCAs were performed for men and women. From the scree plots, three PCs were selected for men and women as they accounted for most of the variance and adding extra PCs did not account for extra variance (online supplemental figure 1). In women, PC1 captured the greatest variance (21.3%) (online supplemental figure 1A) and was driven by central fat (WC, SAT, android fat, and trunk fat) as shown by the factor loadings in figure 3A and online supplemental table 1. This was followed by PC2 (9.8%) (online supplemental figure 1A), driven by serum lipids (TG, TC, and LDL) and liver enzymes (AST, GGT, and ALT). The major contributors of PC3 (8.0%) (online supplemental figure 1A) in women were steroid hormones (androstenedione, estradiol, cortisol, cortisone, and DHEA).

In men, PC1 (27.0%) captured the most variance (online supplemental figure 1B) and the major contributors were peripheral fat (gynoid fat, leg fat) as shown by the factor loadings in figure 3B and online supplemental table 1. This was followed by PC2 (10.6%) (online supplemental figure 1B), driven by liver enzymes (AST, GGT, and ALT) and steroid hormones (androstenedione, cortisol, cortisone, and DHEA). Serum lipids (TG, TC, and HDL) and peripheral fat (gynoid fat, leg fat, and hip circumference) were the major contributing factors for PC3 (6.5%) as shown in figure 3B and online supplemental table 1.

### Association of T2D PRS and risk factors with PPG

In women, the null model including all covariates (age, menopausal stage, smoking status, alcohol consumption, blood pressure medications, lipid medication, HIV status, total physical activity, the total amount of sleep, and asset index) accounted for 14.1% (p=0.035) of the variability in PPG. Model 1 contributed an additional 10.8% to the variability and only PC1 (central fat) and PC2 (lipids and liver enzymes) were significantly (p<0.001 and p=0.003) associated with PPG (table 2). The addition of T2D PRS (Model 2) to the null model did not contribute significantly to the variance (p=0.050) and T2D PRS was not associated with PPG (p=0.564). Accordingly, when adding both T2D PRS and risk factors (Model 3) to the null model, only PC1 (central fat) and PC2 (lipids and liver enzymes) were significantly associated with PPG (p<0.001 and p=0.005) and the model did not account for any additional variability above that which was explained by the risk factors, as reported in Model 1 (10.9% of the variance).

**Table 2:** Multivariable regression associations between sex hormones, liver enzymes, and cardiometabolic factors represented as PCs, PRS, and PG in women (n=269)

Variables	Null model Model R^2^=0.141 Model p value=0.035			Model 1 Model R^2^=0.249 Model p value <0.001			Model 2 Model R^2^=0.142 Model p value=0.051			Model 3 Model R^2^=0.250 Model p value <0.001		
	β	95% CI	P value	β	95% CI	P value	β	95% CI	P value	β	95% CI	P value
**Risk factors**												
PC1				**0.377**	**0.171 to 0.581**	**<0.001**				**0.378**	**0.173 to 0.584**	**<0.001**
PC2				**0.448**	**0.145 to 0.750**	**0.003**				**0.436**	**0.130 to 0.742**	**0.005**
PC3				0.249	−0.099 to 0.599	0.160				0.259	−0.092 to 0.612	0.147
PRS							0.139	−0.336 to 0.615	0.564	0.124	−0.330 to 0.580	0.588

Note: Null model: All the covariates (age, smoking status, alcohol consumption, blood pressure medications, lipid medication, HIV status, total physical activity, the total amount of sleep, and asset index) were included. Model 1: All the covariates with the sex hormones, liver enzymes, and cardiometabolic factors (PC1, PC2, and PC3). Model 2: The covariates and PRS. Model 3: The covariates+sex hormones, liver enzymes, and cardiometabolic factors+PRS.

Bold values are risk factors that are significant in the models.

PC, principal component; PPG, postprandial glucose; PRS, polygenic risk score.

In men, the null model including the covariates contributed an insignificant 4.5% (p=0.398) to the variability in PPG. Model 1 contributed an additional 10.6% to the variability, with PC1 (peripheral fat), PC2 (liver enzymes and steroid hormones), and PC3 (serum lipids and peripheral fat) significantly associated with PPG (p<0.001, p<0.001, and p=0.033, respectively) (table 3). T2D PRS (Model 2) did not add significantly to the null model (p=0.453). When T2D PRS and risk factors were combined in Model 3, these factors explained 11.1% of the variance, and only PC1(peripheral fat), PC2 (liver enzymes and steroid hormones), and PC3 (serum lipids and peripheral fat) remained significantly associated with PPG (p<0.001, p<0.001, and p=0.035, respectively) (table 3).

**Table 3:** Multivariable regression associations between sex hormones, liver enzymes, and cardiometabolic factors represented as PCs, PRS and PPG in men (n=348)

Variables	Null model Model R^2^=0.045 Model p value=0.398			Model 1 Model R^2^=0.151 Model p value <0.001			Model 2 Model R^2^=0.050 Model p value=0.589			Model 3 Model R^2^=0.161 Model p value <0.001		
	β	95% CI	P value	β	95% CI	P value	β	95% CI	P value	β	95% CI	P value
**Risk factors**												
PC1				**−0.342**	**−0.516 to −0.168**	**<0.001**				**−0.348**	**−0.522 to −0.173**	**<0.001**
PC2				**0.475**	**0.210 to 0.742**	**<0.001**				**0.488**	**0.220 to 0.755**	**<0.001**
PC3				**−0.346**	**−0.652 to −0.040**	**0.027**				**−0.342**	**−0.647 to −0.003**	**0.028**
PRS							0.143	−0.232 to 0.518	0.453	0.233	−0.123 to 0.590	0.198

Note: Null model: All the covariates (age, smoking status, alcohol consumption, blood pressure medications, lipid medication, HIV status, total physical activity, the total amount of sleep, and asset index) were included. Model 1: All the covariates with the sex hormones, liver enzymes, and cardiometabolic factors (PC1, PC2, and PC3). Model 2: The covariates and PRS. Model 3: The covariates+sex hormones, liver enzymes, and cardiometabolic factors+PRS. Bold values are risk factors that are significant in the models.

PC, principal component; PPG, postprandial glucose; PRS, polygenic risk score.

## Discussion

This is the first study to evaluate the contribution of genetic and risk factors to PPG variability in a continental African cohort. PCs representing risk factors were significantly associated with PPG variability in both sexes. While a similar amount of the variance in PPG was explained by the anthropometric, biochemical measures, sex hormones, and cardiometabolic factors in both men and women (10.6% vs 10.8%), the factors themselves, represented as PCs, were quite different in men and women. In women, the PCs driven by central fat distribution, serum lipids, and liver enzymes explained most of the variance in PPG, while in men, liver enzymes and steroid hormones-driven PCs were associated with higher PPG, while peripheral fat and serum lipids were associated with lower PPG. The PRS for T2D was not associated with PPG variability in either the men or the women.

In this study, the central fat-driven PC which explained a significant amount of the variance in PPG in women was expected as it has been reported that the accumulation of central fat is linked to glucose intolerance.^28^ Similarly, studies in South Africa have shown that obesity, particularly central obesity as measured by WC and VAT, is a risk factor for developing T2D among urban South Africans of African ancestry women.^29 30^ Moreover, central fat distribution is linked to insulin resistance, fatty liver, and dyslipidemia in women of African ancestry.^31 32^ Indeed, we found that serum lipids and liver enzymes were also associated with PPG. Elevated liver enzymes are a marker of a fatty liver, which is also typically associated with dyslipidemia in adults.^33^ Large studies in the USA have shown that liver fat is lower in individuals of African ancestry than in their European counterparts;^34^ however, data from SA have shown that for a given amount of liver fat, women with SA African ancestry were more insulin resistant than women with SA European ancestry.^35^ Interestingly, this is the first study in a continental African population to report a direct correlation between liver enzymes and PPG, a risk factor for T2D. It is possible to hypothesize that it could reflect fatty liver and further studies are required to measure the association with metabolic dysfunction-associated steatotic liver disease.

In contrast to women, we showed that the elevated peripheral body fat-driven PC was associated with lower PPG in men. Peripheral body fat distribution is more “favourable” as it is associated with reduced insulin resistance, and risk for diabetes and cardiovascular disease, possibly by acting as a sink for excess fatty acids.^36^ The association between PPG and peripheral fat in our study is consistent with the finding by Kufe *et al*,^26^ who showed that higher leg fat mass was associated with higher insulin sensitivity and a 79% lower risk for T2D in men with African ancestry. Further, lipid measures (HDL, LDL, and TC) were associated with lower PPG levels. A recent Mendelian randomization study in people of African ancestry found elevated HDL in men to exert a protective effect against T2D by associating with improved PPG measures.^37^ In contrast, elevated LDL and TC were associated with dyslipidemia and increased T2D risk.^37^ These lipids are known to diminish insulin sensitivity by altering the pancreatic β-cell function.^38^ In this study, we identified an association between serum lipids (HDL, LDL, and TC) and lower PPG levels. The mechanism mediating this observation is not known, but we hypothesize that the glucose metabolism may be linked to the hydroxymethylglutaryl-coenzyme A (HMG-CoA)-reductase activity. HMG-CoA reductase, an enzyme involved in the synthesis of cholesterol, is important for maintaining the cell membrane integrity. Therefore, altered HMG-CoA reductase activity may impact the cell membranes’ composition and fluidity, potentially influencing cellular processes, including glucose metabolism. However, further research is required to verify this.

We also showed in this cohort of middle-aged men that the steroid hormone (cortisol, cortisone, DHEA, and androstenedione)-driven PC was associated with higher PPG. High cortisol concentrations are linked to a high risk for T2D because cortisol regulates glucose metabolism, muscle, and adipose tissues in the liver by increasing gluconeogenesis and decreasing glycogenolysis (breakdown of glycogen) in skeletal muscle.^39^ DHEA and androstenedione are precursors of sex hormones in men and women, and studies on their role in T2D risk are scarce.^40^ A recent European study involving a healthy population of men and women found no association between DHEA and androstenedione levels and T2D incidence.^41^ Instead, the study suggested that DHEA may offer a protective role against T2D because DHEA is a peroxisome proliferator-activated receptor (PPAR) α agonist, which when bound by a PPARα ligand can reduce the onset of T2D. These findings are difficult to explain and require further investigation. There have been a few studies on testosterone levels (a well-established risk factor for T2D) and dimorphic associations with T2D in men and women.^42^ However, this did not show up in our analysis.

Polygenic risk scores have been used for risk stratification in large populations, and for selected diseases they could assist in diagnosis, and refine risk management and treatment strategies.^43^ In a previous UK study, the genetic factors explained 48% of the variance in PPG.^2^ The study used GWAS specific to PPG, was performed in twin studies, and comprised younger healthy adults of European ancestry.^2^ In contrast, our study which used T2D PRS found no associations with PPG variability in South African men and women of African ancestry without T2D. A T2D PRS was used as this was derived from a GWAS in African-origin populations and there is no PRS specific to PPG in Africans. Alternatively, other studies have examined the association between PPG and specific genes. For example, the *TSPAN8* gene has been implicated in glucose homeostasis, and some studies suggest associations with PPG levels without a strong link to T2D.^44^ In addition, in Asian and European ancestry populations, certain variants of the *TCF7L2* gene have been associated with an increased risk of impaired glucose metabolism and high levels of PPG.^45^ However, the variants in this gene may not be applicable to people of African ancestry due to differences in linkage disequilibrium and allele frequencies. Thus, understanding the specific genetic factors associated with PPG in diverse populations such as those of African ancestry may contribute to a more accurate and representative understanding of the genetic basis of PPG.

### Limitations and strength

Strengths of the study include the detailed phenotyping of the participants, including DXA measures of body composition and fat distribution, detailed biochemical measures, and steroid hormones measured using mass spectrometry. Our study is among the first to use clustering on such an extensive range of phenotypic data. However, future studies should include the gut microbiome and other multi-omics approaches, which have been found to be associated with PPG variability.^2^

Our study also has limitations. The study was a cross-sectional design from which we cannot infer causality and thus, we are uncertain about the direction of the associations. We did not make use of the longitudinal analyses because the OGTT and the detailed phenotyping were only performed at one-time point. Lastly, the African American population that was used to generate the PRS differs genetically from continental Africans and this might have contributed to the limited predictive power of the T2D PRS. It is also a limitation that the genetic architecture for T2D is not directly linked to that of PPG.

### Conclusion

The novel approach of using data from middle-aged South Africans of African ancestry demonstrated that inter-individual differences in PPG responses to an OGTT may be differentially explained by body fat distribution, serum lipids, liver enzymes, and steroid hormones in men and women. Our findings also show that there are still unknown factors contributing to inter-individual variability. Understanding the factors associated with the PPG variability from identical meals will help in precision medicine approaches for people in Africa. Hence, multi-omic studies are required to identify other contributing factors to the variability in populations with African ancestry.

## Data Availability

Data are available in a public, open access repository. Data are available upon reasonable request. The dataset used in this study is available in the European Genome-phenome Archive (EGA) database (https://ega-archive.org/) under the study accession code EGAS00001002482. The genotype dataset accession code is EGAD00010001996. The availability of these datasets is subject to controlled access through, the Data and Biospecimen Access Committee of the H3Africa Consortium. The augmented MASC data are available upon reasonable request.
