# Supplementary material for: Postprandial glucose variability and clusters of sex hormones, liver enzymes, and cardiometabolic factors in a South African cohort of African ancestry
Source: BMJ Open Diabetes Res Care. 2024 Mar 7;12(2):e003927. doi: 10.1136/bmjdrc-2023-003927 (PMC10921533; doi:10.1136/bmjdrc-2023-003927)
Supplement: Supplementary data [file bmjdrc-2023-003927supp001.pdf]

A. Supplemental material – List of Figures

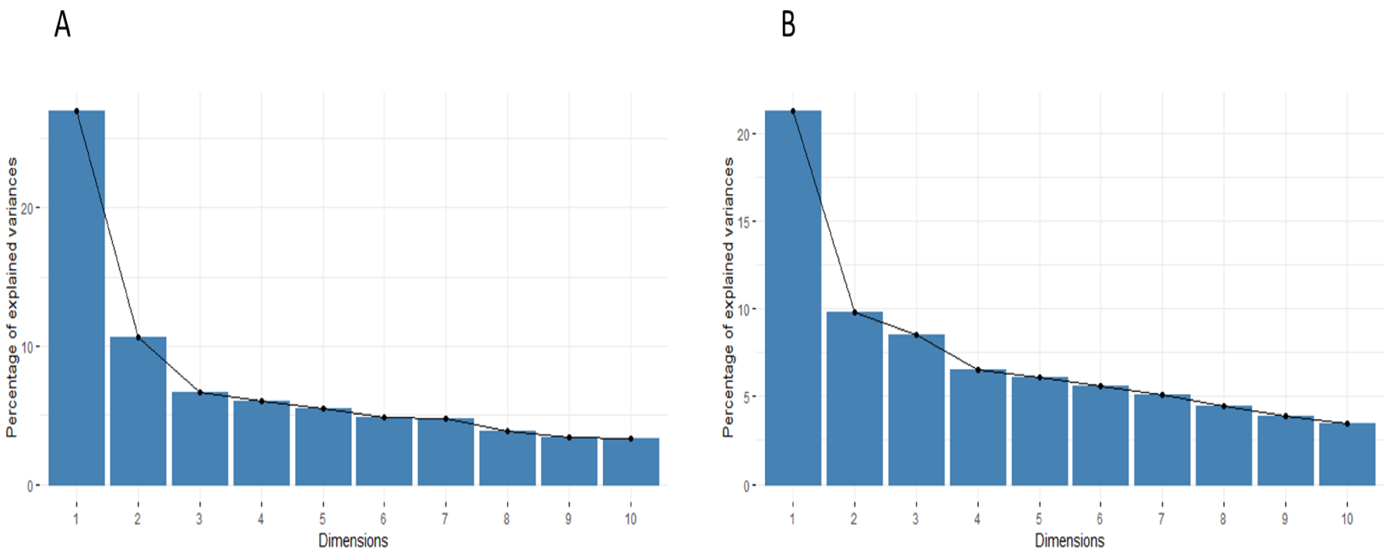

Supplemental figure 1: Scree plots for women (A) and men (B) showing the distribution of principal components by their variance contribution to PPG.

B. Supplemental material – List of Tables

Supplemental table 1: Factor loadings of the major contributing principal components in women and men.

	Women			Men		
**Metabolic factors**	**PC1**	**PC2**	**PC3**	**PC1**	**PC2**	**PC3**
WC (cm)	**0.329**	-0.038	0.028	-0.318	-0.004	0.081
SAT (cm^2)	**0.322**	-0.116	-0.073	-0.306	0.024	0.058
Weight (kg)	**0.321**	-0.220	-0.107	-0.304	-0.065	0.164
Body fat (%)	**0.308**	-0.218	-0.145	-0.308	-0.022	0.115
VAT (cm^2)	0.145	0.064	-0.018	-0.285	0.120	-0.049
FFSTM (kg)	**0.295**	-0.19	-0.046	-0.268	-0.090	0.180
Android (%FM)	**0.280**	0.167	0.093	-0.252	0.201	-0.167
HC (cm)	0.226	-0.260	-0.149	-0.298	-0.079	**0.162**
Trunk (%FM)	**0.247**	0.108	0.149	-0.210	0.261	-0.251
TG (mmol/L)	0.138	**0.213**	0.103	-0.086	**0.178**	-0.123
DBP (mmHg)	0.083	0.053	-0.001	-0.108	0.156	-0.046
Arm (%FM)	0.081	0.068	0.089	0.131	-0.101	-0.156
SBP (mmHg)	0.063	0.070	-0.000	-0.126	0.133	-0.063
Free testosterone (pmol/L)	0.059	0.054	**0.158**	0.052	0.091	0.083
LDL (mmol/L)	0.053	**0.237**	0.013	-0.093	0.065	**0.410**
Height (cm)	0.045	-0.146	-0.073	-0.024	-0.049	0.259
Androstenedione (nmol/L)	0.031	-0.070	0.430	0.142	0.283	0.040
TC (mmol/L)	0.030	**0.263**	0.050	-0.062	**0.200**	**0.426**
DHEA (nmol/L)	0.027	-0.034	**0.230**	0.046	**0.192**	0.065
GGT (µKat/L)	0.021	**0.209**	0.041	0.041	0.299	0.116
ALT (µKat/L)	0.001	**0.187**	-0.063	0.032	0.269	0.058
Cortisol (pmol/L)	-0.016	-0.010	**0.258**	0.142	**0.294**	-0.006
AST (µKat/L)	-0.026	**0.210**	0.005	0.075	0.254	0.076
Cortisone (pmol/L)	-0.039	0.005	**0.273**	0.091	**0.208**	0.098
Estrogen (pmol/L)	-0.048	-0.193	**0.352**	-0.009	0.109	-0.148
FSH (pmol/L)	-0.052	0.037	-0.350	0.032	0.117	0.093
LH (pmol/L)	-0.056	**0.227**	-0.255	0.055	0.142	0.088
HDL (mmol/L)	-0.123	**0.246**	0.059	0.135	**0.183**	**0.269**
SHBG (nmol/L)	-0.164	-0.119	0.104	0.170	0.082	-0.001
Leg (% FM)	-0.23	-0.304	-0.163	**0.185**	-0.247	**0.309**
Gynoid (% FM)	-0.239	-0.215	-0.134	**0.182**	-0.203	**0.204**

Abbreviations: PC, principal component; SAT, subcutaneous adipose tissue; VAT, visceral adipose tissue; TG, triglycerides; TC, total cholesterol; LDL, low-density lipoprotein cholesterol; HDL, high-density lipoprotein cholesterol; ALT, alanine transaminase; AST, aspartate transaminase; GGT, gamma-glutamyl transferase; LH, luteinising hormone; FSH, follicle-stimulating hormone; DHEA, dehydroepiandrosterone; HC, hip

circumference; WC, waist circumference; DBP, diastolic blood pressure; SBP, systolic blood pressure; FFSTM, Fat–free soft tissue mass; SHBG, sex hormone-binding globulin. *Note*: those in bold are the major contributing risk factors

Supplemental table 2: Comparing OGTT data and no OGTT data .

Variable	With OGTT data	Without OGTT data	P-value
n (%)	794 (77.1)	236 (22.9)	-
**Socio-demographic characteristics**			
Age (years)	53.0 (48.0 – 58.0)	55.0 (51.0 – 60.0)	<0.001
women (n (%))	359 (45.2)	134 (56.8)	<0.001
men (n (%))	435 (54.8)	102 (43.2)	<0.001
**Lifestyle characteristics**			
Smoking tobacco (n (%))	365 (46.0)	69 (29.2)	<0.001
HIV Positive (n (%)))	100(12.6)	16(6.8)	0.422
Alcohol intake (n (%))			0.002
No alcohol intake	309 (38.9)	154 (65.3)	
Alcohol intake (monthly or less and 2-4 times a month)	264 (33.3)	47 (20.1)	
Alcohol intake (2-3 times per week or more)	220 (27.8)	6 (14.6)	
**Body composition measure**			
BMI (kg/m^2)	28.5 (22.8 – 33.1)	34.7 (30.6 – 39.4)	<0.001

Values are expressed as mean±sd, median (25th – 75th percentile), or n (%). Abbreviations: BMI, body mass index
